# Semaphorin 4A as novel regulator and promising therapeutic target in rheumatoid arthritis

**DOI:** 10.1186/s13075-015-0846-4

**Published:** 2015-11-06

**Authors:** Svetlana P. Chapoval

**Affiliations:** SemaPlex LLC, 3632 Chateau Ridge Drive, Ellicott City, MD 21042 USA; Department of Microbiology and Immunology, Center for Vascular and Inflammatory Diseases, University of Maryland School of Medicine, 800 West Baltimore Street, Room 301, Baltimore, MD 21012 USA

## Abstract

Rheumatoid arthritis (RA) is a systemic autoimmune disease manifesting in joint destruction. The recognized hallmark of RA pathogenesis is the involvement of immune cells which produce many mediators potentiating an inflammatory environment. RA synovial fibroblasts (RASFs) contribute significantly to disease progression by initiating and regulating many pathways of joint destruction. Detailed molecular insights into RASF biology may lead to identification of important therapeutic targets. The discovery of common molecular targets for joint resident and inflammatory cells may help to develop the most effective therapeutic strategy. One such pathway includes semaphorin 4A as reported in a recent article in *Arthritis Research & Therapy*.

Rheumatoid arthritis synovial fibroblasts (RASFs) produce a variety of cytokines, chemokines, and matrix metalloproteinases (MMPs) which potentiate the effects of inflammatory and immune cells in rheumatoid arthritis (RA). In a recent article in *Arthritis Research & Therapy*, Wang and colleagues [1] report new findings on semaphorin 4A (Sema4A) in disease. They examined the expression and function of Sema4A in synovial tissues and in RASFs obtained from patients with a confirmed RA diagnosis.

Sema4A belongs to a family of glycoproteins that were originally found in the nervous system and that function as axon guidance molecules. The pioneering semaphorin immunology research by Kumanogoh and colleagues [2] identified Sema4A expression on antigen-presenting cells in the lymphoid tissues. They demonstrated that Sema4A acts as a co-stimulator of T-cell activation in vitro and potentiator of antigen-specific T-cell generation in vivo [2, 3]. In vitro, Sema4A-Fc increased antigen-dependent T-cell proliferation and interleukin (IL)-2 production whereas anti-Sema4A Ab inhibited allogeneic response in T cell–dendritic cell mixed lymphocyte reactions. Sema4A interacts with T-cell immunoglobulin and mucin domain-2 (Tim-2) predominantly expressed on T helper (Th)2 cells [2]. The recent study by Delgoffe and colleagues [4] stressed a critical role of Sema4A-neuropilin-1 interaction in regulatory T (Treg) cell differentiation, survival, stability, and function. Additionally, Sema4A engages several Plexin family members—namely Plexin D1, Plexin B1, and Plexin B2—expressed on non-immune (epithelial or endothelial or both) and immune cells (reviewed in [5]). The multiple and complex ways that Sema4A interacts with its receptors have been shown to play important roles in several physiological and pathological conditions (reviewed in [5]). Targeted disruption of Sema4A in animal models led to impaired antigen-specific T-cell generation and cytokine production [2]. The augmented severity of systemic allergic inflammatory responses was found in ovalbumin-treated Sema4A-deficient mice and was associated with increased Treg cell numbers, whereas Th1/interferon-gamma response was not affected [6, 7]. Rennert and colleagues [8] have shown that Tim-2-deficient mice exhibited increased lung inflammation and Th2 cytokine production in response to allergen. Hence, the opposite effects of Sema4A in autoimmune and allergic inflammatory diseases suggest that Sema4A promotes Th1 cell and suppresses Th2 cell responses. However, recombinant human Sema4A (rhSema4A) effectively inhibited already established allergic lung inflammation [9], and bone marrow chimeric mice demonstrated an equal importance of Sema4A expression on lung resident cells and bone marrow-derived inflammatory cells for optimal disease manifestation [8]. Thus, the function of Sema4A in autoimmune and allergic diseases is complex and affects many immune and non-immune cells.

Wang and colleagues [1] demonstrated significantly higher levels of Sema4A mRNA and protein expression in synovial tissues of RA patients as compared with those with osteoarthritis. Moreover, the levels of secreted Sema4A positively correlated with disease activity score. Invasive ability of RASF was potentiated by rhSema4A and blocked with Sema4A small interfering RNA (siRNA). Pro-invasion activity of Sema4A was supported by the data on Sema4A-dependent induction of MMP-3 and MMP-9 expression in RASFs. Furthermore, there is a positive stimulatory Sema4A-nuclear factor-kappa-B (NF-κB) loop as the silencing of p50 or p65 with siRNA constructs led to inhibition of Sema4A expression and rhSema4A treatment upregulated NF-κB phosphorylation in cells. In addition, rhSema4A-dependent IL-6 production in RASFs was attenuated by a specific NF-κB inhibitor. The induction of IL-6 by rhSema4A in RASFs was significantly inhibited by silencing Plexin B1 and partially inhibited by silencing Plexin D1 and TIM-2, thus clearly demonstrating the different levels of involvement of three Sema4A receptors in RA pathogenesis relative to RASF activation and function. The lipopolysaccharide-induced release of other proinflammatory cytokines, namely tumor necrosis factor-alpha and IL-1β, in TH1 cell line was significantly promoted by rhSema4A. Importantly, all three cytokines are considered to be the critical regulators of RA pathogenesis and their inhibition had demonstrated the reverse effects on disease progression and significant improvement of disease activity scores [10]. In addition to all of the above, the detected overexpression of soluble Sema4A in RA patients’ synovial fluids and serum may prove the potential of Sema4A as a diagnostic and prognostic marker for disease initiation, progression, and therapeutic intervention.

Taken together, the data presented by Wang and colleagues [1] pave the way for further investigations of Sema4A in autoimmune diseases, including RA, and provide evidence for its therapeutic potential. The multifaceted activity of Sema4A in coordination of numerous immunologic and pathologic mechanisms involved in RA requires their careful and detailed individual examination.

## Abbreviations

IL: Interleukin
MMP: Matrix metalloproteinase
NF-κB: Nuclear factor-kappa-B
RA: Rheumatoid arthritis
RASF: Rheumatoid arthritis synovial fibroblast
rhSema4A: Recombinant human semaphorin 4A
Sema4A: Semaphorin 4A
siRNA: Small interfering RNA
Th: T helper
Tim-2: T-cell immunoglobulin and mucin domain-2
Treg: Regulatory T
